# Correction: Proteomic mapping of cytosol-facing outer mitochondrial and ER membranes in living human cells by proximity biotinylation

**DOI:** 10.7554/eLife.50707

**Published:** 2019-08-05

**Authors:** Victoria Hung, Stephanie S Lam, Namrata D Udeshi, Tanya Svinkina, Gaelen Guzman, Vamsi K Mootha, Steven A Carr, Alice Y Ting

Hung V, Lam SS, Udeshi ND, Svinkina T, Guzman G, Mootha VK, Carr SA, Ting AY. 2017. Proteomic mapping of cytosol-facing outer mitochondrial and ER membranes in living human cells by proximity biotinylation. *eLife* **6**:e24463. doi: 10.7554/eLife.24463.

Published 25, April 2017

Recently while revisiting our own protocols, we became aware of two typographical errors in our Materials and Methods section. Under the section entitled "Preparation of EM samples related to SYNJ2BP overexpression in wild type or RRBP1 knockout cells with or without protein translation inhibitors", we incorrectly stated the reagent used to reduce OsO_4_ .

Below is the original text:

"Each well was then overlaid with 1 ml of a 1:1 mixture of 2% K_3_[Fe(CN)_6_] in sodium cacodylate buffer:2% OsO_4_ (Electron Microscopy Sciences) solution in cold sodium cacodylate buffer for 2 hr."

And the corrected version:

"Each well was then overlaid with 1 ml of a 1:1 mixture of 2% K_4_[Fe(CN)_6_] · 3H_2_O in sodium cacodylate buffer:2% OsO_4_ (Electron Microscopy Sciences) solution in cold sodium cacodylate buffer for 2 hr."

Additionally, in the methods section entitled "Western blot analysis of SYNJ2BP-V5 pull downs in the presence of translation inhibitors", we incorrectly recorded the western blot protocol for detecting endogenous anti-SYNJ2BP rather than the correct protocol used to detect the V5 epitope-tagged recombinant SYNJ2BP as clearly denoted in Figure 4A.

Below is the original text:

"The bottom halves were blocked in 3% BSA in 1x TBST, and the top halves were blocked in 5% milk in 1x TBST for 1 hr at room temperature. The top halves were incubated face down on 500 µl of 1:3000 rabbit anti-RRBP1 (Abcam, catalog no. ab95983) in 1% milk in 1x TBST for 1 hr, and the bottom halves were incubated face down on 500 µl of 1:500 rabbit anti-SYNJ2BP (Sigma Aldrich, catalog no. HPA000866) for 1 hr. The membranes were then washed four times with 1x TBST for 5 min each time. Each membrane was rocked in 10 ml of 1:2000 anti-rabbit HRP (Bio-rad) in 3% BSA in 1x TBST each for 1 hr at room temperature."

And the corrected version:

"The bottom halves were blocked in 3% BSA in 1x TBST, and the top halves were blocked in 5% milk in 1x TBST for 1 hr at room temperature. The top halves were incubated face down on 500 µl of 1:3000 rabbit anti-RRBP1 (Abcam, catalog no. ab95983) in 1% milk in 1x TBST for 1 hr. The bottom halves were each rocked in 1:5000 mouse anti-V5 (Life Technologies, catalog no. R960-25) in 10 mL of 3% BSA in 1xTBST at room temperature for 1 hr. The membranes were then washed four times with 1x TBST for 5 min each time. Each membrane was rocked in 10 ml of either 1:2000 anti-rabbit HRP (Bio-rad) or 1:2000 anti-mouse HRP (Bio-rad) in 3% BSA in 1x TBST for 1 hr at room temperature."

These corrections do not affect any of the scientific findings or conclusions of the original paper, but we hope they will increase clarity. We apologize for any confusion these errors may have caused.

The article has been corrected accordingly.

